# Jottings in Operative Dentistry

**Published:** 1878-02

**Authors:** Jas. F. Thompson.

**Affiliations:** Fredericksburg, Va.


### ARTICLE III.

### *Jottings in Operative Dentistry.*

BY JAS. F. THOMPSON., D. D. S., FREDERICKSBURG, VA.

[Read before the Virginia State Dental Association, Dec. 12, 1877.]

As chairman of the Committee on Operative Dentistry, I have felt it my duty to make a report of some sort to this Association, and as the time at my command has not allowed me to write an elaborate thesis on any one subject, I have thought that instead of a more extended effort in one field, I would make a few jottings of ideas occuring in relation to subjects directly connected with practice, trusting I may be excused for the somewhat desultory character of this hastily prepared paper.

I commence with a subject, the discussion of which has filled volumes.

Let us take our stand beside our chair armed with all the nerve force which any operation may call for, for dentists should have a good share of nerve force and to spare; for who has not felt at the end of an hour or two of work over a nervous patient that exhaustion which signals excessive loss of nerve force? while perhaps only a slight fatigue would be experienced in dealing with a patient who was calm and quiet under our manipulations.

I am at a loss to attribute this effect to any other cause than want of sympathy between the patient and the operator, and in such a case would it not be well to dismiss our patient for the time, and take some other day, trusting that another sitting we may find him or her less exhausting to our nerves? For we must feel that it is well nigh impossible for us to perform perfect operations for our patients whilst in this condition of non-sympathy with them. Indeed so far true is this, that many of us are willing to confess that there is a difference in the quality of our work at different times of the day. Who has not felt in the morning satisfied in the performance of operations, which in the afternoon were beyond his ability?

But perhaps the most annoying trouble—I had almost said the greatest curse—the dentist has to deal with is sensitive dentine. Nothing in all my experience wearies me so soon as a sensitive tooth, and the question as to how we shall manage such cases as require immediate operation upon, without time for the trial of such of the numerous paleiatives as are used as temporary stoppings. For with us whose practise is largely on country patients, it is almost imperative that the operation should be completed at once, I have tried the almost innumerable remedies which have been vaunted from time to time as specifics for sensitive dentine, with but slight effect for good, one only remains to be tested—the extract of eucalyptus—and I shall try that so soon as I can get hold of the drug. The best treatment however I have found to be moral courage and sharp instruments, but these signally failed me a short while ago in the case of a very nervous patient with the most hypersensitive tooth I have ever seen. Chloride of zinc, "dental pain obtunder," aconite, iodine, all utterly failed to give any relief. The lady had heart disease, else I should have given chloroform, as she was obliged to leave town, when it occured to me to use, or to try atmospheric anæsthesia, the latest of the methods of anæsthesia and the suggestion of Dr. Bonwill, of Phila. Accordingly instructing her in the methods of breathing, she proceeded to inhale deeply for a minute or so, and I then proceeded to excavate the cavity, requesting her to keep up the profound respiration, when astonishing as it may seem, I was enabled to remove the decay and prepare and fill the cavity without special trouble.

I do not propose to discuss the subject of filling teeth, but as to the material to be used in filling, premising that we have carefully prepared the cavity, something may be said. When I am allowed, I always prefer gold, and use this under all circumstances in the anterior teeth, and I advise my patients that in the end they will find it the cheapest. A few times I have used tin for anterior teeth, but I tell you that I have lost patients by refusing to use amalgam in such cases. As to forms or styles of gold I prefer the semi cohesive, and non-cohesive. I have found that these forms of gold when condensed can be added to with annealed foil, and so the filling finished with these. I consider that they cohere as perfectly as though melted and run together, provided the cavity is perfectly dry. I am disposed to think soft foil the most reliable of the various forms of gold, as it adapts itself more perfectly to the irregularities of the cavity than any other form of foil. It is also more easily condensed laterally, pressing out against the walls and thus filling the tooth more perfectly.

And now I will tell you of something which I have successfully tried, which possibly some here present have not, and that is tin foil in combination with gold foil. I am aware that this contact of two different metals in the same cavity is contra-indicated by that bugbear galvanic action. But I have patients in whose mouths are fillings of this compound character, which they assure me have been in the grinding surfaces of these teeth for twenty-five years. The other teeth in the same mouths which had been filled with gold were badly decayed, but these were perfectly sound. To the touch of the excavator these fillings sound almost as hard as steel, and I could plainly see the different layers or alternating folds of metal. As these fillings had succeeded so well, I was determined to try this for myself, and so the first opportunity that offered I filled two large grinding surface cavities with tin and gold folded together, about two-thirds gold being used. When finished these fillings seemed about of the hardness of ordinary tin fillings. I saw them a few days ago, and they had undergone some sort of crystalline change, the character of which I am not able to explain, but they were as hard as amalgam, and preserving the teeth perfectly. I confess I should like for some one able to do so, to tell me how it is that this change occurs in these metals in combination, and what the character of the change is.

As to plastic fillings I do not hesitate to say that I use them in preference to tin, and by plastic I mean that "nasty stuff," which some fine haired dentists would not touch, but which I call amalgam. You will have some of these fine gentry stand up at associations and say that next to gold they would use tin foil, and nothing else, but if you could take a peep behind the scenes you would find a small cup of amalgam already prepared for use—on the sly. I have however the honor of the acquaintance of a gentleman, a most worthy member of this association, who when I last talked with him had never used amalgam, and I give him the credit of saying that I believe his statement to be true, though I am sure he has lost money by it. The best amalgam I have used is the "standard alloy," made by Eckfeldt and Dubois, of Philadelphia. I will conclude my remarks on amalgam by saying that I recently saw a filling of this material—a large one—which had effectively preserved a tooth for thirty years.

In finishing fillings we all find the dental engine an indispensable instrument now-a-days. It is not however so useful in proximal as in crown fillings. For the former I have been for some time in the habit of using emery tape, as advised by Dr. M. H. Webb, but I find of late that fine sand-paper is better than the emery and has the merit of being cheap. It has performed this part of the work more satisfactorily than anything of the kind I have used.

The removal of salivary calculus is a more important operation than is usually esteemed by practitioners. I

do not think we have to deal with a more destructive disease than the inflammation produced around the necks of the teeth by tartar. The peculiar disease attendant upon this combination of affairs called "Riggs' disease," from the fact I suppose that that gentleman has been so successful in its treatment, seems best managed by a surgical operation on the alveolus, removing the border of dead or dying bone with instruments of a peculiar shape, some of which I here exhibit.

It is hardly worth while for me to touch the subject of replantation of teeth. It is a practice I have looked for. ward with much interest to its being brought to perfection. At a previous meeting of this association the subject was discussed by several members and their various success given. I have seen some cases of long years standing as proofs of its practicability,—one I am familiar with in the mouth of the late lamented Preston Welford, M. D., who so nobly gave his life a martyr to duty, dying of the yellow fever at Fernandina, whither he had gone as a volunteer physician. The tooth in question was extracted and replaced in 1854, and was in service at the time of Dr. Welford's death. And I have the record of another case—an old lady of 82, who said she had extracted a tooth forty odd years ago, for a negro man, a lower jaw tooth at that, and getting out the wrong tooth,—a sound one,— she put it back again, and it grew strong and solid and remained a useful organ to the day of his death.

The last scene which ends the strange and eventful history of our teeth, or rather those of our patients, for however we may haul out theirs, we are shy of loosing are own, —extraction. We all know that this is an operation as much calculated to unnerve a patient almost, as any other upon the human body, and every agency that science can call to its aid for the purpose of alleviating the pain incident to this operation should be used. It is through the practice of our noble and humane profession that the blessings of nitrous oxide and ether, which last paved the way for chloroform, have been given to mankind. In bringing these hastily collected jottings to a close I feel that they would be imperfect, did I not mention a method of local anæsthesia which I can best give in the words of the newspaper from which I cut it:—

A Painless Operation in Dentistry.—Dr. R. E. Moore tried an experiment on a patient at Dr. Farmer's office a few days since which was successful in the object sought, and should excite more attention than it has heretofore. The patient seated himself to have a tooth drawn—a tooth very tight in the jaw, and much difficulty was overcome in extracting it. To relieve the pain attending the extraction Dr. Moore placed his thumbs on both temples of the patient, and we have it from the gentleman's lips that, although the tooth was broken off and the roots extracted with difficulty, the pain was almost entirely destroyed by this pressure. The theory of this we cannot fully explain, but it will present itself at once to the informed of medical and dental professions.

